# First Report of *Cowpea Mild Mottle Carlavirus* on Yardlong Bean (*Vigna unguiculata* subsp. *sesquipedalis*) in Venezuela

**DOI:** 10.3390/v4123804

**Published:** 2012-12-14

**Authors:** Miriam Brito, Thaly Fernández-Rodríguez, Mario José Garrido, Alexander Mejías, Mirtha Romano, Edgloris Marys

**Affiliations:** 1 Vegetal Virology and Phytopathogenic Bacteria Laboratory, Institute of Agricultural Botany, Agronomy Faculty, Central University of Venezuela, ZIP 4579, Maracay 2101-A, Venezuela; E-Mails: britom@agr.ucv.ve (M.B.); mariojgarrido@gmail.com (M.J.G.); 2 Biotechnology and Plant Virology Laboratory, Center for Microbiology and Cell Biology, Venezuelan Institute for Scientific Research (IVIC), ZIP 20632, Caracas 1020-A, Venezuela; E-Mails: thaly.fernandez-rodriguez@agroscience.rlp.de (T.F.-R.); amejias@ivic.gob.ve (A.M.); mirtha.romano@gmail.com (M.R.)

**Keywords:** *Vigna unguiculata*, *sesquipedalis*, yardlong bean, *Cowpea mild mottle carlavirus*, CPMMV, diagnosis, RT-PCR, Venezuela

## Abstract

Yardlong bean (*Vigna unguiculata* subsp. *sesquipedalis*) plants with virus-like systemic mottling and leaf distortion were observed in both experimental and commercial fields in Aragua State, Venezuela. Symptomatic leaves were shown to contain carlavirus-like particles. RT-PCR analysis with carlavirus-specific primers was positive in all tested samples. Nucleotide sequences of the obtained amplicons showed 84%–74% similarity to corresponding sequences of *Cowpea mild mottle virus* (CPMMV) isolates deposited in the GenBank database. This is the first report of CPMMV in Venezuela and is thought to be the first report of CPMMV infecting yardlong bean.

## 1. Introduction

*Cowpea mild mottle virus* (CPMMV) was first reported as causing systemic mottling, chlorotic blotches and leaf malformations in cowpea (*Vigna unguiculata* L.) in 1973 in Ghana [1]. Since then, it has been subsequently reported from several tropical regions of Africa [2,3,4,5,6], Asia [7,8,9,10], Brazil and Argentina [1,11], and from the Ivory Coast [12]. The experimental host range of CPMMV includes plant species from several families, which mainly display chlorotic local lesions or systemic mottle upon inoculation or infection. CPMMV has flexuous filamentous particles (approx. 650 nm in length) resembling those of members of the genus *Carlavirus*. CPMMV is transmitted in a non-persistent manner by the whitefly *Bemisia tabaci* G. [13], and has also been assigned to the genus *Carlavirus*, currently classified in the family *Betaflexiviridae* [13]. Carlaviruses can reduce yields of some crop species by 10%–15% and, in mixed infections, can exacerbate the deleterious effects of other viruses.

Cowpea is an annual tropical grain legume, which plays an important role in the nutrition of people in developing countries of the tropics and subtropics, especially in sub-Saharan Africa, Central Asia, and South America. Yardlong bean (*Vigna unguiculata* (L.) Walp. subsp. *sesquipedalis *(L.) Verdc.) is a distinctive subspecies of cowpea, with apparent origin in East Asia. Yardlong bean is characterized by extremely long and thin pods, and is considered one of the top ten Asian vegetables. It is now grown extensively in Asia, Europe, Oceania and America [14,15]. Several viruses are known to infect yardlong bean, including *Cucumber mosaic virus* (CMV), *Bean common mosaic virus* (BCMV) and *Mungbean yellow mosaic virus* (MYMV) [16]. In 2011, yardlong bean plants showing mild leaf mottling, mild mosaic and leaf distortion symptoms were found in an experimental plot at the Agronomy Faculty, UCV, in Aragua State, Venezuela. Further surveys in 2012 confirmed the presence of symptoms in commercial yardlong bean fields (Pao de Zárate, Aragua State, Venezuela). Crops were heavily infected with *Bemisia tabaci*. Electron microscope observations by the leaf-dip method showed that symptomatic leaves contained semi-flexuous virion particles, *ca.* 600 nm in length, typical of carlavirus species. We describe here the identification and partial characterization of CPMMV isolated from diseased yardlong bean plants.

## 2. Results and Discussion

**Figure 1:**
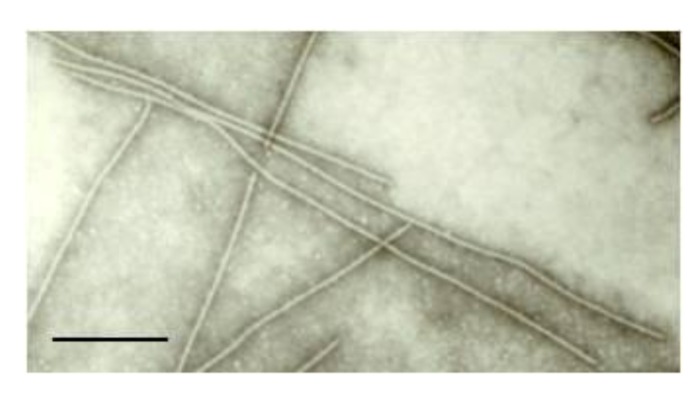
Transmission electron micrograph showing viral particles in sap of symptomatic *V. unguiculata *subsp. *sesquipedalis* leaf. Bar: 100 nm.

The commonest symptoms suggestive of virus infection in a field survey of *V. unguiculata *subsp. *sesquipedalis* were leaf mottling, mild mosaic and slight leaf distortion. Virus incidence ranged from 15% to 40%. Preliminary examination of the extracts of symptomatic leaves in negatively stained preparations under a transmission electron microscope consistently revealed the presence of long, slightly flexuous particles 13 nm wide and 600–700 nm long, within the expected size range for carlavirus (Figure 1). Virus-infected cells were found to contain particles in bundle-shaped aggregates (data not shown), suggesting infection by a carlavirus [17].

**Figure 2:**
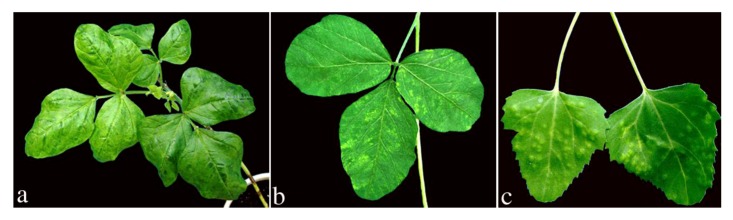
Leaf symptoms in inoculated *V. unguiculata* subsp. *sesquipedalis *(**a**), *G. max *(**b**) and *C. amaranticolor* (**c**).

Mechanical transmission assays resulted in systemic infection of legume plants such as *V. unguiculata* subsp. *sesquipedalis*, *V. unguiculata* subsp. *unguiculata*, *Phaseolus vulgaris* L., *Arachis hypogaea* L. and *Glycine max* (L.) Merr. (Figure 2). Symptoms appeared 10–14 days after inoculation, and the most obvious symptom was mottling and slight leaf deformation. Non-legume plants such as *Chenopodium quinoa *Willd. and *Chenopodium amaranticolor* Coste et Reyn. developed chlorotic local lesions 10–14 days after inoculation (Figure 2). Three weeks after whitefly transmission experiments, *V. unguiculata* subsp. *sesquipedalis* and *V. unguiculata* subsp. *unguiculata* plants showed mottling, mild mosaic and slight leaf deformation symptoms. The virus was transmitted by whiteflies to these host species with an average efficiency of 56% and 52%, respectively. The symptoms induced following mechanical and insect transmission tests to host plants resembling those described for *Cowpea mild mottle virus* (CPMMV), a leguminous seed and whitefly-borne virus [11,18]. 

CPMMV was first reported on cowpea in Ghana [1], and subsequently reported from several tropical regions of Africa [2,3,4,5,6], Asia [7,8,9,10], Brazil and Argentina [19,20], as well as from the Ivory Coast [21]. 

Despite the fact that it has previously been shown that CPMMV is transmitted by seed in soybean [1], the ability of CPMMV to be transmitted via seed is still unclear. Our results clearly showed that CPMMV can be transmitted from yardlong bean seeds contaminated with virus to seedlings. From a total of 231 seeds harvested from previously infected plants, 222 seedlings were obtained and evaluated up to 45 days after emergence, and 40% of the plants developed symptoms of mottling, mild mosaic and slight leaf deformation. The high rate of found seed transmission highlights the risks of using seeds from yardlong bean-infected plants to contribute to the further spread of CPMMV.

**Figure 3:**
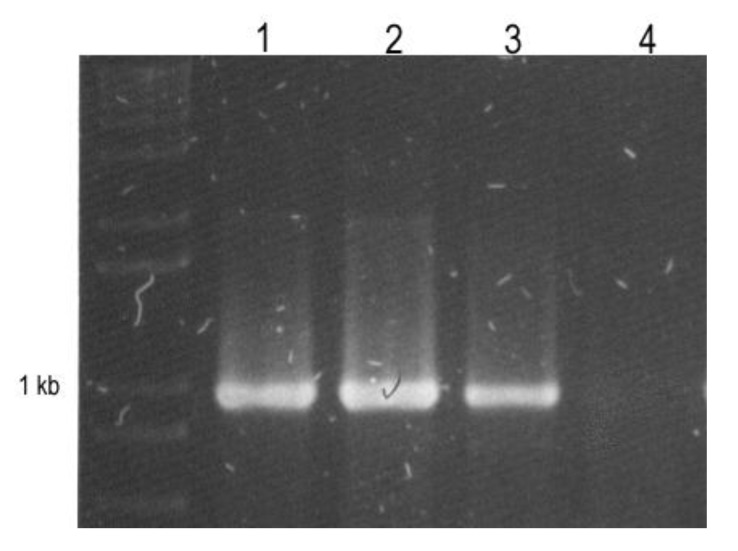
RT-PCR detection of *Cowpea mild mottle virus *(CPMMV) in infected (1–2) yardlong bean samples, positive CPMMVcontrol (3), negative (healthy) control (4).

Sequence analysis has become one of the most important criteria for classification. The semi-flexuous-shaped particles observed in symptomatic yardlong bean plants suggested that the isolated virus was a carlavirus. Moreover, the host range, symptomatology induced in test plants, and transmission via seeds and by whiteflies, all agreed well with what has been reported for CPMMV [1,11,18,22], but the amplification of a *ca.* 0.9 bp fragment using carlavirus universal primers [13,21] was the most convincing argument for concluding that the virus isolated from yardlong bean is a carlavirus (Figure 3). 

**Figure 4:**
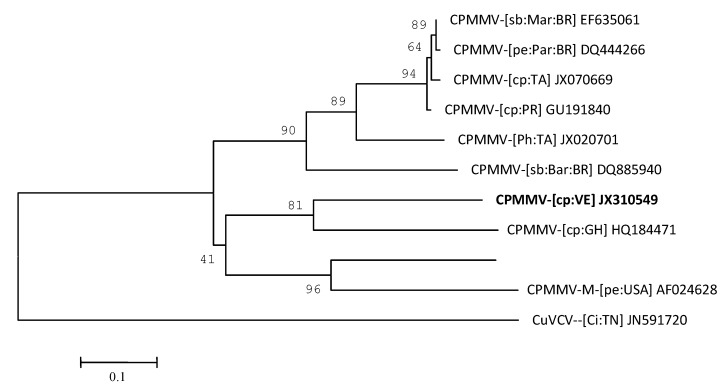
Phylogenetic tree based on partial sequence of coat protein gene and complete sequence of putative nucleic acid binding protein gene of isolates of CPMMV. Bootstrap values (1,000 replicates). Virus abbreviations and NCBI accession numbers are as follows: Cowpea mild mottle virus-[soybean:Maranhao:Brazil] (CPMMV-[sb:Mar:BR] EF635061), CPMMV-[peanut:Parana:Brazil] (CPMMV-[pe:Par:BR] DQ444266), CPMMV-[cowpea: Puerto Rico] (CPMMV-[cp:PR] GU191840), CPMMV-[soybean:Barreiras:Brazil] (CPMMV-[sb:Bar:BR] DQ885940), CPMMV-[cowpea: Taiwan] (CPMMV-[cp:TA] JX070669), CPMMV-CY-[ Phaseolus: Taiwan] (CPMMV-[Ph:TA] JX020701), CPMMV-[ cowpea:Venezuela] (CPMMV-[cp:VE] JX310549), CPMM-M-[peanut:USA] (CPMMV-M-[pe:USA] AF024629), CPMM-H-[peanut:USA] (CPMMV-M-[pe:USA] AF024628), CPMMV-[cowpea:Ghana] (CPMMV-[cp:GH] HQ184471). Cucumber vein-clearing virus-[Citrullus:Tanzania] (CuVCV--[Ci:TN] JN591720) was used as outgroup.

The 3' genomic region was selected for analysis due to the high efficiency of the universal carlavirus primers [21]. The nucleotide sequence of the DNA fragment amplified with degenerate primer pairs M4T and Carla-CP was determined to be 958 bp (accession No.: JX310549). A BLAST (Basic Local Alignment Search Tool) analysis for sequence similarity of the CP gene and the 3' UTR confirmed the identity of the virus, sharing the highest identity with CPMMV isolated from *V. unguiculata *subsp. *unguiculata* in Ghana (CPMMV-[cowpea:Ghana] (CPMMV-[cp:GH] HQ184471) at 84%, and with CPMMV isolates from *G. max* in Brazil (Cowpea mild mottle virus-[soybean:Maranhao:Brazil] (CPMMV-[sb:Mar:BR] EF635061) and from *Arachis repens* in Parana State, Brazil (CPMMV-[peanut:Parana:Brazil] (CPMMV-[pe:Par:BR] DQ444266) at 74%. The percentage of sequence similarity with CPMMV, suggests that the virus from yardlong bean is an isolate of this species. Phylogenetic analysis of the putative nucleic acid binding protein and CP aa sequences (Figure 4) demonstrates that CPMMV isolate clusters together with all other CPMMV isolates, forming one clade with the bean (HQ184471) isolate from Ghana and soybean (EF635061) isolate from Brazil.

## 3. Experimental

### 3.1. Virus Source and Biological Assays

Yardlong bean plants showing disease symptoms suggesting viral origin were collected in an experimental plot at the Agronomy Faculty, UCV, in Aragua State, Venezuela. Leaf samples showing symptoms of mottling, mild mosaic and slight leaf distortion were brought to UCV and IVIC laboratories, examined by electron microscopy and subjected to mechanical inoculation assays to *V. unguiculata *subsp. *sesquipedalis* and *V. unguiculata *subsp. *unguiculata*, *Phaseolus vulgaris* cv. Top Crop, *Solanum lycopersicum*, *Arachis hypogaea* L., *Glycine max* (L.) Merr, *C. quinoa* and *C. amaranticolor*. Non-viruliferous whiteflies (*B. tabaci*) biotype B were maintained in *Canna indica *and given an access period of 24 h on infected yardlong bean plants. Groups of ten insects were transferred to healthy *V*. *unguiculata* subsp. *sesquipedalis* (71 plants) and *V*. *unguiculata* subsp. *unguiculata* cv. Tuy (21 plants), and left to feed for 48 h, then killed by spraying an insecticide. Tests evaluations were done at seven-day intervals. For test of seed transmission, yardlong bean plants were infected in the cotiledonary stage, and seeds were sown on flats in a greenhouse. Infection was determined visually and verified by back inoculations on *C. amaranticolor*. Symptomatic seedlings were further evaluated for virus presence by RT-PCR.

### 3.2. Virus Particle and Cytopathology

Using standard procedures, crude sap from symptomatic plants was stained with 2% phosphotungstic acid, pH 7.0, and ultrathin sections of infected leaves were stained with 1% uranyl acetate and lead citrate before examination with a Philips CM10 electron microscope (Phillips Company, Eindhoven, The Netherlands).

### 3.3. Reverse Transcription and RT-PCR Detection.

Total RNA was extracted from 0.1 g fresh (or 0.02 g dried) leaf samples with the RNeasy Plant Mini Kit (Qiagen, Hilder, Germany) according to the manufacturer’s protocol. First-strand cDNA was synthesized using SuperScript^TM^ III Reverse Transcriptase (Invitrogen, Carlsbad, CA, USA) according to the manufacturer’s instructions and with M4T (5'-GTT TTC CCA GTC ACG ACA C (T)16-3') as the initial primer [23]. The PCR reaction contained 50 ng template cDNA, 10 pmol of each amplification primer Carla-CP (5'-GGB YTN GGB GTN CCN CAN GA-3') [21] and M4T, 200 mM each dNTP, 1.25U Taq DNA polymerase (Invitrogen), 5 mM MgCl_2_, 50 mM KCL, 20 mM tris-HCL pH 8.3 in 25 μL. Amplification parameters were: initial denaturation at 94 °C for 5 min and 35 cycles of 94 °C for 1 min, 50 °C for 1 min, and 72 °C for 2 min, followed by final extension at 72 °C for 10 min. Amplification products were examined by electrophoresis in 1% agarose gels containing ethidium bromide and visualized using the UV transilluminator. Amplified products were purified with the QIAquick PCR purification kit (Qiagen Inc., Chatsworth, CA, USA) and ligated into a pGEM-T Easy vector (Promega, Madinson, WI, USA) and competent *Escherichia coli* cells (JM-109) were transformed by following standard molecular biological procedures. Four recombinant clones from the same PCR product were sent to Macrogen Inc. (Seoul, Korea) for sequencing. For each sample, a consensus sequence was obtained using BioEdit software [24]. Then, a mega BLAST search was performed. The phylogenetic tree was inferred using MEGA5 with 1,000 replicates of the neighbor joining procedure [25].

## 4. Conclusions

Our experimental data clearly point out that the yardlong bean plants with mottling and mosaic symptoms found in the Aragua state of Venezuela were infected by an isolate of CPMMV. Possibly, it was transmitted by whiteflies present in the crop. This is the first report of CPMMV naturally occurring in yardlong bean, and the first record of CPMMV in Venezuela. However, the possibility of latent CPMMV infections in other crops cannot be ruled out. High mutation rates could facilitate host range changes that may eventually lead to epidemics. 

A survey of the plants growing near a yardlong bean field must be made in order to identify possible natural reservoirs of the virus. Also, a thorough screening for resistance to CPMMV on the available *Vigna* germplams collection is necessary. It is not yet known how the virus came into the country, but its spread can be also explained by the international trading of infected seed. The presence of CPMMV in Venezuela must be considered as a threat to this crop, as well as to other legume crops, since whitefly populations are present everywhere. This report will form the basis for further molecular characterization of the CPMMV isolate.
